# Influence of Vinyl Polysiloxane Impression Techniques on Marginal Fit of Metal Frameworks for Fixed Partial Dentures

**DOI:** 10.3390/ma13204684

**Published:** 2020-10-21

**Authors:** Joseph Nissan, Ofir Rosner, Gal Rosen, Sarit Naishlos, Eran Zenziper, Helena Zelikman, David Lavi, Liat Chaushu

**Affiliations:** 1 Department of Oral Rehabilitation, The Maurice and Gabriela Goldschleger School of Dental Medicine, Tel Aviv University, POD 39040 Tel-Aviv, Israel; rosnerop@yahoo.com (O.R.); ggrosen@gmail.com (G.R.); eranzen@gmail.com (E.Z.); helenapl@gmail.com (H.Z.); lavidav@yahoo.com (D.L.); 2 Departments of Pedodontology, School of Dental Medicine, Tel Aviv University, POD 39040 Tel-Aviv, Israel; river554@gmail.com; 3 Department of Periodontology and Implant Dentistry, School of Dental Medicine, Tel Aviv University, POD 39040 Tel-Aviv, Israel; liat.natanel@gmail.com

**Keywords:** vinyl polysiloxane, impression techniques, marginal fit accuracy

## Abstract

Impression technique is one of the factors affecting restoration fit accuracy, which is a major aspect influencing its survival. The purpose of this study is to compare, in vivo, the effect of two commonly used Vinyl Polysiloxane (VPS) impression techniques on the metal framework fitting of fixed partial dentures. Ninety-two consecutive patients, diagnosed as partially edentulous, treated by fixed partial denture restorations, participated in the study. Group 1-impressions (*n* = 44) were subjected to the 1-step technique, while group 2 impressions (*n* = 48) were subjected the 2-step technique. Three accuracy assessment common methods: probe, tactile sense and radiographic test, were used to validate the clinical fit of the metal framework. Misfit was defined as even one test failure. Twenty-one (22.8%) out of 92 metal frameworks exhibited metal frameworks misfit, whereas the other 71 (77.2%) were found to be accurate. Group 1 presented significantly (*p* = 0.04) more metal frameworks misfit, 14/44 (31.8%) vs. 7/48 (14.6%). Restoration location (maxilla vs. mandible) had no statistically significant impact on the results (*p* = 0.461). The use of the VPS putty/wash 2-step impression technique is recommended to improve the clinical fit of fixed partial denture restorations.

## 1. Introduction 

Restoration fit accuracy is an important predictor which determines the survival of fixed partial dentures. Discrepancy between restoration and the prepared tooth may influence periodontal health, plaque retention, bone resorption, caries rate, and pulpal pathology [1,2,3,4,5]. Impression is one of the major factors controlled by the clinician that may influence the fit accuracy of the restoration [6,7,8,9,10]. The impression phase is mostly affected by the impression materials and techniques [11,12,13,14,15,16,17,18,19,20,21]. Even with technology progress and increased use of computer analysis such as 3D image analysis systems, vinyl polysiloxane (VPS) is still widely used as an impression material for fixed partial denture fabrication, due to its high degree of detail reproduction, dimensional stability, and low cost [22,23,24,25,26].

The putty-wash impressions can be made using several techniques [11,12,13,14,15,16,17,18,19,20,21]. Despite the fact that some publications justified the use of a one-step impression technique [25,26], many others reported that the two-step with controlled wash bulk was found to be the most accurate [17,18,19,20,21,22]. A wash-bulk thickness of up to 2 mm seems to be an important factor for fabricating an accurate stone dies working model, using VPS impression materials [18,27]. However, there is little information regarding the association between these putty-wash impression techniques and restorations’ marginal fit accuracy [28,29]. The importance and uniqueness of the present in vivo study is that most of the previously reported data originated from in vitro studies. The purpose of the present retrospective study was to compare, in vivo, the effect of two commonly used VPS impression techniques on the metal framework fit accuracy of fixed partial dentures. The working hypothesis was that the two-step impression technique with controlled wash bulk would be more accurate.

## 2. Materials and Methods

### 2.1. Enrollment

Consecutive partially edentulous patients with a treatment plan of 3–4 units fixed partial denture restorations in the posterior (distal to the canines) jaw area (45% mandible, 55% maxilla) were included in the study. The study population consisted of 92 individuals (42 women and 50 men), aged 49 ± 16 years. A total of 92 fixed partial denture metal frameworks (9—4 in group 1 and 5 in group2—out of 92 with 4 units and 83/92 with 3 units) were examined: 47 in the maxilla and 45 in the mandible. Teeth preparations were made by prosthodontists, residents in Oral Rehabilitation or under their supervision, at the Department of Oral Rehabilitation, School of Dentistry, Tel-Aviv University. All procedures were fully explained to the patients who signed an informed consent, and the Ethics Committee of the Tel Aviv University approved the study protocol. This research did not receive any specific grant from funding agencies in the public, commercial, or not-for-profit sectors.

### 2.2. Impression Techniques

**Figure 1:**
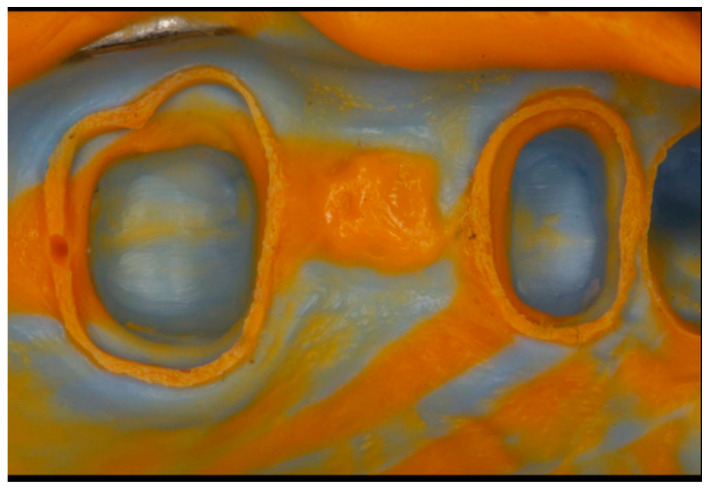
One-step impression technique.

All the impressions were taken by a single experienced prosthodontist (JN) with perforated metal stock trays (Medesy) and processed by either of the following impression techniques, randomly chosen by the prosthodontist preference: For group 1 (Figure 1), impressions were subjected to the 1-step technique, in which putty and wash impression materials were used simultaneously. The wash material was dispensed with an automatic mixing syringe. 

**Figure 2:**
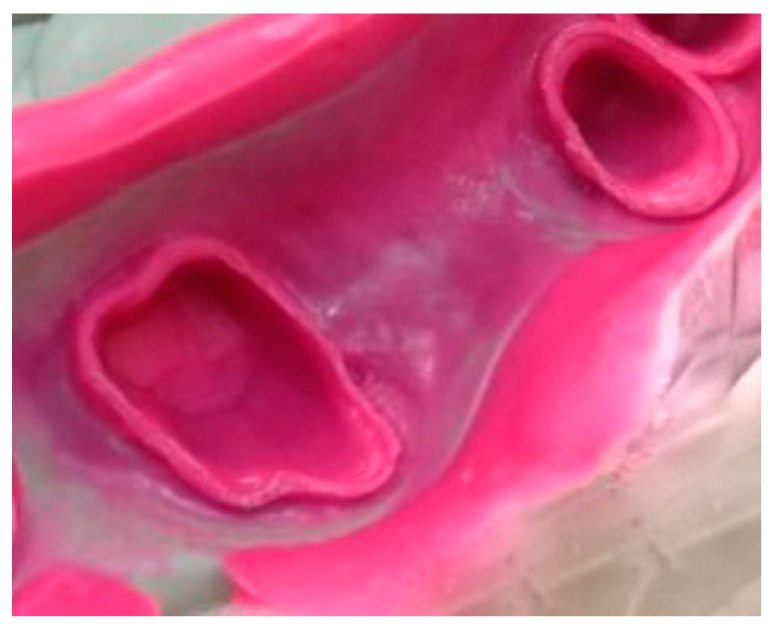
Two-step impression technique.

For group 2 (Figure 2), impressions were subjected to the 2-step technique, in which the putty impression was performed first and allowed to set for 6 min. After setting, putty was removed from the prepared abutments sites using a round low-speed bur nr. 16 (Dentsply DeTrey Gmbh, Charlotte, NC, USA),in order to create about 2 mm space for wash material [13], which was injected on the prepared teeth and into the putty using an automatic mixing syringe. The impression was then reseated and allowed to set. 

A double chord technique was used to open the gingival sulcus and present the margin. Impressions were made with VPS impression material (3M-ESPE) at viscosities of putty (Express STD ISO 4823 type 0) and light body (Express-Regular set light body hydrophilic ISO 4823 type 3). All materials were mixed in standardized proportions and were measured with plastic cups according to the manufacturer’s recommendations. The tray adhesive supplied by the manufacturer was applied evenly over the tray’s surface. Impressions were poured in improved die stone (type IV, Supra stone; Kerr, Sybron, Brea, CA, USA). All materials were mixed in standardized proportions according to the manufacturers’ recommendations. Setting time was conducted by the manufacturer’s recommendation. All impressions were disinfected before pouring. All impressions were stored at room temperature (25 °C) for 1 h before pouring assuring a similar effect of humidity on the setting of the impression material. The improved stone was first mixed by hand to incorporate the water and then mechanically mixed under vacuum for 15 s. All mixes were vibrated into the impressions and allowed to set for 1 h before separation. The metal framework was produced using Argelite 60+ alloy (59.9% Pd, 26.3% Ag, Argen Corporation, San Diego, CA, USA). A single laboratory (Shenhav Ltd.) and a single technician was responsible of fabricating the restorations.

### 2.3. Accuracy Verification

A single experienced prosthodontist (JN) verified accuracy of all fixed partial dentures in the patient’s mouth. According to the department’s policy, three common clinical accuracy assessment methods were used for the metal tray before finishing the crown to validate clinical fit of all restorations metal framework [30,31]: probe test (using a dental probe for verifying vertical and horizontal restorations marginal fitting); tactile sense test (“rocking test” for verifying the restoration metal framework stability) and radiographic test (bitewing radiograph verifying interproximal restorations adaptation). Failure was defined as misfit of one of the accuracy assessment methods. All the data were collected from patients’ files and charts and were statistically analyzed.

## 3. Results

Twenty one (22.8%) out of 92 metal frameworks presented misfit, whereas the other 71 (77.2%) were found to be accurate. Group 1 (*n* = 44) presented 14 (31.8%) metal frameworks misfit, while in group 2 (*n* = 48) only 7 (14.6%). (Table 1).

Fisher’s Exact Test showed statistically significant differences between the two impression techniques (*p* = 0.04). Restoration location (maxilla vs. mandible) had no statistically significant impact on the results (*p* = 0.461).

## 4. Discussion

Marginal fit accuracy of a cast restoration is one of the factors that determines its long-term survival. Marginal misfit could lead to coronal microleakage, secondary caries, and plaque accumulation resulting in gingival inflammation, initiating loss of the restored tooth [1,2,3]. Accurate detail registration during impression taking is a crucial factor determining restoration fit and durability.

Previous in vitro studies [17,18,19,20,21] comparing working models accuracy following impressions showed significant differences (*p* = 0.04) between the two impression techniques, in favor of the 2-stage technique. 

In the present study, the accuracy of two common impression techniques, 1-step vs. 2-step, using VPS, was assessed using three common methods to check the metal frameworks marginal fit. The 1-step impression technique led to twice as many metal frameworks misfit, compared to the 2-stage impression technique (31.8% vs. 14.6%). It can be speculated that the critical factor influencing the 2-step putty/wash impression technique accuracy was the wash material bulk control. Ideal thickness was found to be < 1–2 mm [18,27]. Its absence in the 1-step technique (and in the polyethylene technique using nylon or cellophane spacer) leads to differential contraction of the wash material bulk, yielding uneven dimensional changes. 

Similarly, another in vitro study evaluated the framework marginal fit (nondestructive technique) and showed that the two-stage impression technique was the most accurate method for VPS impression taking [28,29].

In vivo challenges such as presence of saliva, tongue, and floor of mouth movement did not affect the impression technique accuracy and emphasize the superiority of the 2-stage technique.

Although the 1-step technique has the advantages of being easier and faster, the putty tends to push the wash away from the preparation, and thus important areas, such as the finish line, may be recorded by the putty, which has been shown to be less precise [11,12,13,14,15,16,17,18,19,20,21,22,23]. Furthermore, dimensional changes (vertical, horizontal) occurred due to impression material contraction towards the tray walls resulting in wider and shorter stone dies at the working model [32,33,34,35,36,37]. In the 2-step technique, the wash stage is carried out after the putty has set and contracted, and served as a custom tray. Furthermore, tray rigidity may affect the accuracy of polyvinyl siloxane impressions. Plastic trays produced less accurate impressions than metal trays [38]. The controlled wash bulk compensates for this contraction with minimal dimensional changes. The present in vivo results demonstrated the superiority of the 2-step impression technique in terms of restoration accuracy while using custom metal trays. Further in vivo studies are needed to determine the accuracy of the impression technique in the intraoral environment.

## 5. Conclusions

Within the limitations (retrospective, one center, one impression material) of the present in vivo study, it can be concluded that the VPS putty/wash 2-step impression technique produced a more accurate metal framework for fixed partial denture restorations compared to the 1-step impression technique.

## Figures and Tables

**Table 1:** Clinical result for impression technique.

Impression Technique	N	Misfit	Fit
**One-step**	**44**	**14 (31.8%)**	**30 (68.2%)**
**Two-step**	**48**	**7 (14.6%)**	**41 (85.4%)**
**Total**	**92**	**21**	**71**
